# Height Compensation Using Ground Inclination Estimation in Inertial Sensor-Based Pedestrian Navigation

**DOI:** 10.3390/s110808045

**Published:** 2011-08-15

**Authors:** Sang Kyeong Park, Young Soo Suh

**Affiliations:** Department of Electrical Engineering, University of Ulsan, Namgu, Ulsan 680-749, Korea; E-Mail: damiro76@hotmail.com

**Keywords:** inertial sensors, angle measurement, pedestrian navigation, Kalman filter

## Abstract

In an inertial sensor-based pedestrian navigation system, the position is estimated by double integrating external acceleration. A new algorithm is proposed to reduce *z* axis position (height) error. When a foot is on the ground, a foot angle is estimated using accelerometer output. Using a foot angle, the inclination angle of a road is estimated. Using this road inclination angle, height difference of one walking step is estimated and this estimation is used to reduce height error. Through walking experiments on roads with different inclination angles, the usefulness of the proposed algorithm is verified.

## 1. Introduction

A pedestrian navigation system provides a person’s location indoors or outdoors. Many different technologies are used for the pedestrian navigation such as vision [1], wireless technology [2], ultrasonic sensors [3,4], and inertial sensors [5–10]. Among them, an inertial sensor-based pedestrian navigation system computes the location using inertial sensors installed on a shoe [5–10]. This inertial navigation system can be used for first respondents and soldiers [11].

The inertial-based navigation algorithms can be classified into two groups. One is that a step length is estimated using some parameters such as one step walking time or accelerometer maximum values [5,6]. The other is that the position is computed using the inertial navigation algorithm [12], where results in [7–10] belong to this group. Basic inertial pedestrian navigation algorithms are proposed in [7,8]. In [9], inertial navigation algorithm is combined with a map matching algorithm. In [10], an HMM filter is used to estimate gait phases and detect zero velocity intervals. Also we note that the inertial navigation algorithm is also used to analyze gait for medical purposes [13,14].

This paper is related to the latter, where the inertial navigation algorithm is used. A main advantage of an inertial sensor-based algorithmis that no landmarks or devices need to be installed on the environment. On the other hand, an inertial navigation algorithm has an inherent problem that the position error grows as time elapses.

To reduce the error growth, all inertial-based algorithms employ the zero velocity updating [15]. During walking, a foot touches the ground almost periodically for a short time interval and we know the velocity of a foot during the interval is zero. This interval is called a zero velocity interval. The errors of the inertial navigation algorithm are reduced using this zero velocity interval.

However, even with the zero velocity, the error growth can be significant. Experiment results reveal that *z* axis position (height) error growth could be in particular significant (see left plots in Figure 8 and Figure 9). One explanation is that acceleration in the *z* axis could be very large when a foot touches the ground and *z* axis acceleration data could be saturated (see Figure 4). This will degrade the position accuracy of all three axes and the effect is most dominant in the *z* axis position and velocity since the acceleration is the largest in the *z* axis direction.

In this paper, we propose a new algorithm, which reduces the *z*-axis position error. We use the fact that a foot angle is almost the same as the inclination angle of a ground when a foot is on the ground (see Figure 2). If we measure a foot angle during the zero velocity (that is, when a foot is on the ground), the inclination angle of a ground can be estimated. Using the estimated inclination angle, the *z*-axis position error can be compensated.

The paper is organized as follows. In Section 2, basic equations of inertial sensor-based pedestrian navigation algorithm is introduced and detailed equations are given in Appendix. In Section 3, a height compensation algorithm using a foot angle estimation is proposed. In Section 4, the proposed algorithm is verified through experiments. Conclusion is given in Section 5.

## 2. Inertial Sensor-Based Pedestrian Navigation System

In this section, a brief introduction to an inertial navigation algorithm is given. Details about inertial navigation algorithms can be found in [12,16,17]. The inertial navigation algorithm used in this paper is from [10].

Let *r* ∈ *R*^3^, *v* ∈ *R*^3^ and *q* ∈ *R*^4^ be a position, a velocity and attitude quaternion in the navigation coordinate frame. The navigation coordinate frame has axes with the direction north (*x* axis), west (*y* axis), and the local vertical (up, *z* axis). The body coordinate frame is fixed on the sensor unit. The rotation matrix associated with the quaternion *q* is expressed as *C*(*q*). In the inertial navigation algorithm, *r*, *v* and *q* are estimated from inertial sensors (accelerometers and gyroscopes) and magnetic sensors.

Let ω*_b_*, *a_b_*, *g͂* and *m͂* be defined as follows:

- ω*b* ∈ *R*^3^ : body angular rates
- *a_b_* ∈ *R*^3^ : body acceleration without gravitational acceleration
- *g͂* ∈ *R*^3^ : gravitational acceleration vector in the navigation coordinate frame
- *m͂* ∈ *R*^3^ : earth magnetic field vector in the navigation coordinate frame.

The sensor output equations are given by

(1) $$\begin{array}{l} y_{g}=\omega_{b}+b_{g}+v_{g}\\ y_{a}=C(q)\tilde{g}+a_{b}+b_{a}+v_{a}\\ y_{m}=C(q)\tilde{m}+v_{m} \end{array}$$

where *y_g_* ∈ *R*^3^ is a gyroscope output, *y_a_* ∈ *R*^3^ is an accelerometer output, *y_m_* ∈ *R*^3^ is a magnetic sensor output, *b_g_* ∈ *R*^3^ is gyroscope bias, *b_a_* ∈ *R*^3^ is accelerometer bias, *v_g_* ∈ *R*^3^ is gyroscope sensor noise, *v_a_* ∈ *R*^3^ is accelerometer sensor noise, and *v_m_* ∈ *R*^3^ is magnetic sensor noise.

An indirect Kalman filter is used to estimate *q*, *r* and *v*. In an indirect filter, *q*, *r* and *v* are not directly estimated. Instead *q*, *v* and *r* are first estimated by appropriately integrating or double integrating *y_g_* and *y_a_* and their errors are estimated using a Kalman filter [10]. The indirect Kalman filter equations are given in Appendix.

The following assumptions are made in the paper:

- Walking direction is only forward and no side walking and backward walking are allowed.
- There is no staircase on the walking path

The assumptions are satisfied during normal walking situations: that is, a person walks forward only and stopping is allowed.

## 3. Height Compensation Algorithm

**Figure 1.**
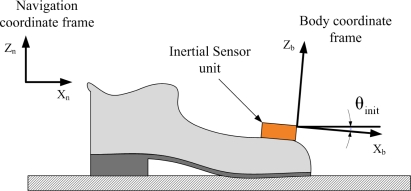
Initial angle *θ_init_* when a foot is on a flat ground.

The inertial sensor unit is installed on top of a shoe as in Figure 1. When a person is standing on a flat ground, the unit is not completely level and the roll angle at that time is denoted by *θ_init_*.

**Figure 2.**
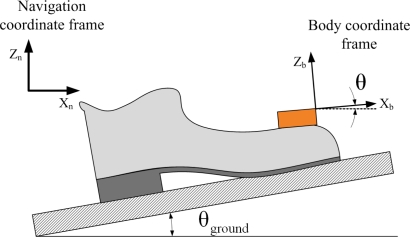
Roll angle *θ* of the sensor unit when a foot is on a slope.

When a foot is on a slope with the inclination angle *θ_ground_* (see Figure 2), the roll angle *θ* of the sensor unit is different from *θ_init_*, which is the roll angle when a foot is on a flat ground. The relationship between *θ* and *θ_ground_* is given by

(2) $$\theta_{\mathrm{groud}}=\theta -\theta_{\mathrm{init}}.$$

Since *θ_init_* is constant, we can estimate the ground inclination angle *θ_ground_* once we know the roll angle *θ*.

The roll angle *θ* can be computed from the attitude quaternion *q̂*. Or *θ* can be also estimated using accelerometer outputs during the zero velocity interval since there is no external acceleration. From Equation (1), we obtain the following ignoring sensor noises and external acceleration

$$y_{a}\approx C(q)\left[\begin{array}{l} \begin{array}{c} 0\\ 0 \end{array}\\ g \end{array}\right]=\left[\begin{array}{l} \begin{array}{c} -\text{sin}\theta \\ \text{cos}\theta \text{sin}\varphi \end{array}\\ \text{cos}\theta \text{cos}\varphi \end{array}\right]g$$

where *φ* is the pitch angle. Thus *θ* and *φ* can be estimated using the following:

(3) $$\begin{array}{lll} \phi & = & \text{atan}2(y_{a,y},y_{a,z})\\ \theta & = & \text{atan}2(-y_{a,x},\;\sqrt{y_{a,y}^{2}\;+\;y_{a,z}^{2}}). \end{array}$$

In this paper, roll angle *θ* is estimated using Equation (3) during each zero velocity interval: *θ* is computed for each discrete time during a zero velocity interval and the averaged value is used as *θ̂*, which is an estimated value of *θ*. Thus *θ̂* is updated whenever zero velocity intervals are encountered. In the update, a low pass filter is used to suppress a sudden change of *θ̂*.

There are many methods to detect zero velocity intervals [15]. In this paper, we used both accelerometer values and force sensors (Tekscan FlexiForce Sensors), which are installed inside a shoe. Note that the measured force increases when a foot is on the ground since the human weight is applied on the sensors. We assume a discrete time *i* belongs to a zero velocity interval if the measured force sensor is larger than the prespecified value and *θ_i_* (*θ* value computed using Equation (3) at the discrete time *i*) satisfies the following

(4) $$|\theta_{i}-\theta_{i-1}|<2^{\circ }.$$

Note that Equation (4) is equivalent to the condition that changes of accelerometers are small.

**Figure 3.**
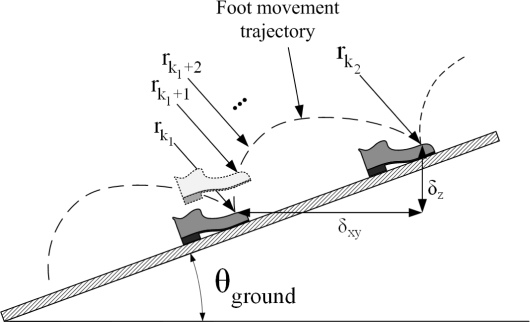
Foot movement between two zero velocity intervals.

A typical foot movement trajectory when a person is walking on a slope is given in Figure 3. Note that *r*_*k*_1__ is the foot position at the discrete time *k*_1_. In this example, the foot is on the slope at the discrete time *k*_1_ and *k*_2_. A person walked one step between the time *k*_1_ and *k*_2_. Thus *r*_*k*_1__ is the position before one step walking and *r*_*k*_2__ is the position after the step. Let *δ_z,_*_*k*_1_,__*k*_2__ and *δ_xy,_*_*k*_1_,__*k*_2__ be the horizontal and vertical distances between *r*_*k*_1__ and *r*_*k*_2__, respectively:

(5) $$\begin{array}{l} \delta_{\mathrm{xy}}={\left\|\left[\begin{array}{l} \begin{array}{ccc} 1 & 0 & 0 \end{array}\\ \begin{array}{ccc} 0 & 1 & 0 \end{array} \end{array}\right](r_{k_{2}}-r_{k_{1}})\right\|}_{2}\\ \;\;\;\delta_{z}=\left[\begin{array}{ccc} 0 & 0 & 1 \end{array}\right](r_{k_{2}}-r_{k_{1}}). \end{array}$$

Note that *δ_z,_*_*k*_1_,__*k*_2__ and *δ_xy,_*_*k*_1_,*k*_2__ are horizontal and vertical distances of one walking step. We will drop *k*_1_ and *k*_2_ subscripts in *δ* for simplicity.

Assuming that walking is mostly up or down along the slope, *δ_z_* and *δ_xy_* have the following relationship:

(6) $$\delta_{z}\approx \text{tan}(\theta_{\mathrm{ground}})\delta_{\mathrm{xy}}.$$

Equation (6) is used in the measurement update of the Kalman filter in Appendix. Let *θ̂*_*k*_1__ be estimated *θ* value at time *k*_1_ using Equation (3) and *r̂*_*k*_1__ be the position estimate of the inertial navigation algorithm. Let ${\hat{r}}_{k_{2}}^{-}$ be the position estimate of the inertial navigation algorithm before the measurement update (that is, the zero velocity updating). Thus ${\hat{r}}_{k_{2}}^{-}$ is obtained by double integrating acceleration starting from time *k*_1_ with the initial value *r̂_k_*__1__.

From Equation (6), let *δ̂_z_* (estimate of δ*_z_*) be defined by

(7) $${\hat{\delta }}_{z}=\text{tan}({\hat{\theta }}_{k_{1}}-\theta_{\mathrm{init}}){\left\|\left[\begin{array}{l} \begin{array}{ccc} 1 & 0 & 0 \end{array}\\ \begin{array}{ccc} 0 & 1 & 0 \end{array} \end{array}\right]({\hat{r}}_{{}_{k_{2}}}^{-}-{\hat{r}}_{k_{1}})\right\|}_{2}.$$

Note that *δ̂_z_* in Equation (7) is the vertical distance (height difference) computed using the ground inclination angle.

We have assumed that *θ̂_k_*__1__ is relatively accurate and errors in *x* and *y* position estimation in the inertial navigation algorithm are small: that is, we have assumed the following is satisfied

$$\left[\begin{array}{l} \begin{array}{ccc} 1 & 0 & 0 \end{array}\\ \begin{array}{ccc} 0 & 1 & 0 \end{array} \end{array}\right]({\hat{r}}_{k_{2}}^{-}-{\hat{r}}_{k_{1}})\approx \left[\begin{array}{l} \begin{array}{ccc} 1 & 0 & 0 \end{array}\\ \begin{array}{ccc} 0 & 1 & 0 \end{array} \end{array}\right](r_{k_{2}}-r_{k_{1}}).$$

With the assumptions we have the following approximation from Equations (5) and (6):

(8) $${\hat{\delta }}_{z}\approx \left[\begin{array}{ccc} 0 & 0 & 1 \end{array}\right](r_{k_{2}}-r_{k_{1}}).$$

Let *v_z_* be the approximation error in Equation (8), we can rewrite Equation (8) as following:

(9) $$\begin{array}{l} {\hat{\delta }}_{z}=\left[\begin{array}{ccc} 0 & 0 & 1 \end{array}\right](r_{k_{2}}-r_{k_{1}})+v_{z}\\ \;\;\;\;\;\;\;=\;\;\;\left[\begin{array}{ccc} 0 & 0 & 1 \end{array}\right](r_{k_{2}}-{\hat{r}}_{k_{2}}^{-}+{\hat{r}}_{k_{2}}^{-}-r_{k_{1}})+v_{z}\\ \;\;\;\;\;\;\;=\;\;\;\left[\begin{array}{ccc} 0 & 0 & 1 \end{array}\right](r_{e,k_{2}}+{\hat{r}}_{k_{2}}^{-}-{\hat{r}}_{k_{1}}-r_{e,k_{1}})+v_{z} \end{array}$$

where recall that $r_{e,k}=r_{k}-{\hat{r}}_{k}^{-}$ in Equation (13).

Let $v_{\delta }=v_{z}-\left[\begin{array}{ccc} 0 & 0 & 1 \end{array}\right]r_{e,k_{1}}$, then Equation (9) can be written as follows:

$${\hat{\delta }}_{z}-\left[\begin{array}{ccc} 0 & 0 & 1 \end{array}\right]({\hat{r}}_{k_{2}}^{-}-{\hat{r}}_{k_{1}})=\left[\begin{array}{ccc} 0 & 0 & 1 \end{array}\right](r_{k_{2}}-{\hat{r}}_{k_{2}}^{-})+v_{\delta }$$

where *v*_δ_ represents the approximation errors.

We have the following measurement equation for the Kalman filter in Appendix:

(10) $${\hat{\delta }}_{z}-\left[\begin{array}{ccc} 0 & 0 & 1 \end{array}\right]({\hat{r}}_{k_{2}}^{-}-{\hat{r}}_{k_{1}})=\left[\begin{array}{ccccccccccccccc} 0 & 0 & 0 & 0 & 0 & 0 & 0 & 0 & 1 & 0 & 0 & 0 & 0 & 0 & 0 \end{array}\right]x_{k_{2}}+v_{\delta }.$$

This measurement update equation is combined with the zero velocity updating equation in Equation (16). Note that the measurement noise *v*_δ_ contains all the approximation errors in the derivation of Equation (10). So an exact analytic formulation for the covariance is not easy to derive. A small positive value is assigned to $E\{v_{\delta ,k_{2}}v_{\delta ,k_{2}}^{\prime }\}$ in the paper.

The proposed algorithm combined with the inertial navigation algorithms summarized in the following:

while (true)
    compute ${\hat{r}}_{k}^{-}$, ${\hat{v}}_{k}^{-}$ and ${\hat{q}}_{k}^{-}$
    if (zero velocity interval)
      if (the start of the zero velocity interval)
        *θ̂_ground_* = *θ̂_ground,previous_*
      else if (the end of the zero velocity interval)
        compute *θ̂_ground,previous_* using Equations (2) and (3)
      end
      zero velocity updating Equation (16) and height compensation Equation (10)
      update *r̂_k_*, *v̂_k_* and *q̂_k_*
    else
      
${\hat{r}}_{k}={\hat{r}}_{k}^{-}$, ${\hat{v}}_{k}={\hat{v}}_{k}^{-}$ and ${\hat{q}}_{k}={\hat{q}}_{k}^{-}$
    end
    *k* = *k* + 1
end

## 4. Experiments

As an inertial sensor unit, XSens MTi28A53G25 is used, whose specifications are given in Table 1.

**Figure 4.**
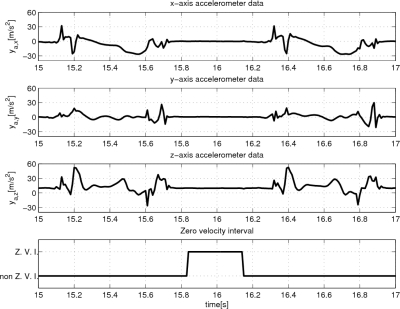
Accelerometer outputs and zero velocity interval.

In Figure 4, typical accelerometer data (*y_a_*) are given. The accelerometer output from XSens MTi28A53G25 is a low pass filtered signal with the bandwidth 30 Hz (see Table 1). The full scale range of the accelerometer is 50 *m*/*s*^2^ and note that there is saturation in *y_a,z_*, which may cause large *z* axis velocity and position errors. The detected zero velocity interval is also given.

**Figure 5.**
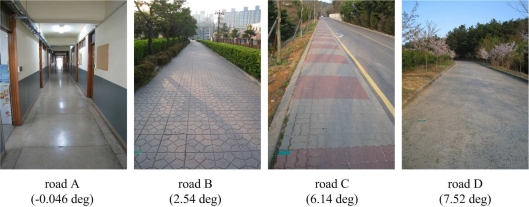
Four roads (A,B,C,D) with different inclination angles.

**Figure 6.**
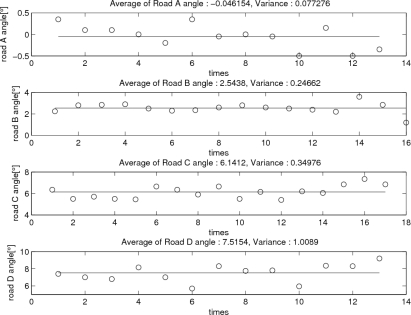
Inclination angle measurement with a digital inclinometer (each measurement is taken from different points along the roads).

To test the proposed algorithm, four roads are selected (see Figure 5). These roads are more than 50 m long and the inclination angles are almost constant. The inclination angle of each road is measured with a digital inclinometer: inclination angles are measured at several points (13–17 points for each road) and the average value is considered as the inclination angle of a road. The results are given in Figure 6.

In Figure 5, the inclination angles of four roads are −0.046°, 2.54°, 6.14° and 7.52°, respectively.

**Figure 7.**
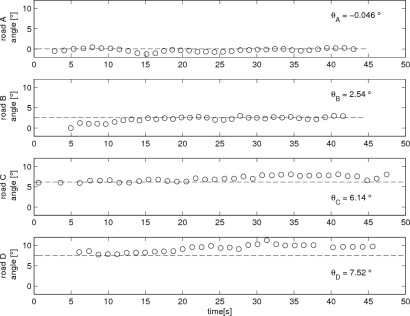
*θ̂_ground_* estimation for each road (the estimated value at the end of a zero velocity interval).

We walked up on each road 50 m and computed *θ* angle using Equation (3) during the angle measurement interval. In Figure 7, computed *θ̂_ground_* using Equations (2) and (3) is given. Note that each point in the figure corresponds to a computed *θ̂_ground_* for each walking step. For the reference, the road inclination angles are also given. We can see that *θ̂_ground_* is close to the inclination angle and thus *θ̂_ground_* can be used as a road inclination angle estimate.

**Figure 8.**
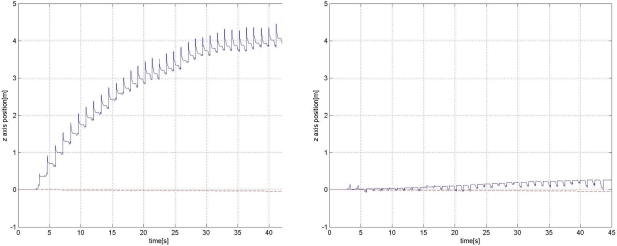
*z* axis position estimation for road A without (left) and with (right) the proposed height compensation.

The *z* axis position estimation (the third element of *r*) result for road A (indoor corridor) is given in Figure 8. The left graph in Figure 8 shows the *z* axis position estimation without the height compensation, where the inertial navigation algorithm with zero velocity updating is used. The straight line in the plot is the estimated actual *z* axis position, which is computed from the inclination angle of the road and the walking distance (50 m). We can see the error increases rapidly. The error growth depends on many elements such as sensor scaling factor calibration, bias stability, sensor axis alignments and sensor saturation. We only performed simple calibrations. An initial gyroscope bias is estimated by averaging initial 1 minute gyroscope data while the sensor unit is not moving. Also, the accelerometer offset is estimated by rotating the accelerometer 360° and finding the center value. With this simple calibration, the error seems to be large. The right graph in Figure 8 shows that the *z* axis position is corrected using the proposed height compensation algorithm.

**Figure 9.**
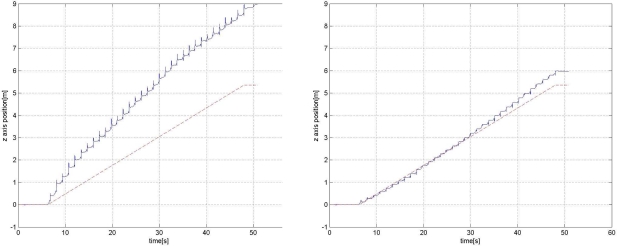
*z* axis position estimation for road C without (left) and with (right) the proposed height compensation (up-walking).

The *z* axis position estimation result for road C (inclination angle of the road is 6.14°) is given in Figure 9, where a person walked up 50 m along the road. The straight line is drawn between 0 and the computed final *z* axis position (50 m × sin(6.14°) = 5.348 m). Note that we measured 50 m using a tape measure on the road and thus 50 m corresponds to ||*r_N_* − *r*_1_||_2_, where *r_N_* is the final position and *r*_1_ is the initial position. Without the height compensation, we can see that the *z* axis position error is large (final *z* axis position error is 3.61 m). On the other hand, with the height compensation, the *z* axis position error compensation is greatly reduced (final *z* axis position error is 0.62 m).

**Figure 10.**
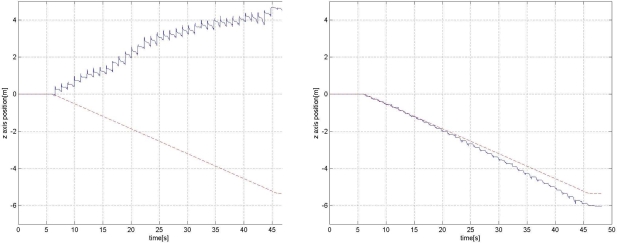
*z* axis position estimation for road C without (left) and with (right) the proposed height compensation (down-walking).

For the same road C, we walked down 50 m along the road and the result is given in Figure 10. It can be seen that without the height compensation, the *z* axis position error diverges quickly. In the right plot, it can be seen that the *z* axis position is compensated with the height compensation algorithm.

For four roads, three walking experiments are done. The average *z* axis position errors are given in Table 2 without and with the compensation algorithm. The true final *z* position is computed using sin(*θ_ground_*) × 50 m. We can see that the proposed height compensation algorithm reduces the *z* axis position error significantly. In Table 2, position errors (with compensation) of Road D seem to be large. We believe this is due to the fact that the road D does not have a smooth surface, which can be verified from Figure 6. Thus the computed true height (that has been estimated using the estimated slope angle 7.52°) may not be accurate.

We note although the proposed method reduces the *z* axis position error growth, the position error divergence cannot be avoided over the long time.

**Figure 11.**
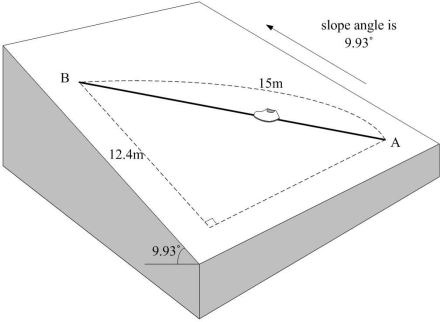
Height compensation experiment while walking up and down the slope diagonally.

Now instead of walking up and down along the slope, a person walked up and down the slope diagonally. In this experiment, pitch angle *φ* is not zero. We measured road inclination angles along line B in Figure 11 and the average road inclination angle is 7.83°. A person walked up and down 3 times. The *z* axis position error with the height compensation was 0.45, 0.43, 0.15 m (walking up) and 0.41, 0.30, 0.21 m (walking down). Thus we can see the proposed algorithm is working when *φ* is not zero.

## 5. Conclusions

In pedestrian navigation systems using inertial navigation algorithm, position error tends to diverge sooner or later. To reduce the position error growth, a zero velocity updating algorithm is used. Even with the zero velocity updating algorithm, position error growth could be still large. In particular, the *z* axis position (height) error growth could be significant.

In this paper, we have proposed a height compensation algorithm. An inclination angle of a road is estimated using foot angle estimation. Using the inclination angle, the height difference of a walking step is estimated. Using this estimation, *z* axis position in the inertial navigation algorithm is compensated. Through walking test under four different roads (with different inclination angles), the usefulness of the proposed method has been shown. Four different roads (with different inclination angle), 50 m walking test was done. Without the proposed height compensation algorithm, the average *z* axis position error range was 1.64–8.94 m over 50 m walking. On the other hand, the average error range with the proposed height compensation algorithm was 0.05–2.11 m.

We note that although the proposed method reduces the *z* axis position error growth, the position error divergence cannot be avoided over the long time. To avoid the divergence problem, external reference such as GPS should be used.

The current algorithm assumes that a person walks up or down on a slope direction. The current algorithm cannot deal with the staircase walking. The future work is to improve the proposed algorithm to cope with various situations such as the staircase walking. One possible solution is to use gait phase information (which can be determined using inertial sensors and force sensors [18]) to determine whether a person is stair climbing or descending.

## Figures and Tables

**Table 1.** Specifications of XSens MTi28A53G25.

	rate of turn	acceleration	magnetic field
full scale	±1, 500°/s	±50 m/ s^2^	±750 mGauss
bandwidth	40 Hz	30 Hz	10 Hz
bias stability	20°/ h	0.02 m/s^2^	0.1 mGauss
noise	0.05°/ s/ $\sqrt{\text{Hz}}$	0.002 m/ s^2^/ $\sqrt{\text{Hz}}$	0.5 mGauss

**Table 2.** *z* axis position error (average value of 3 experiments).

		position error without compensation (m)	position error with compensation (m)
road A		5.76	0.41
road B	walking up	1.59	0.05
	walking down	3.51	0.30
road C	walking up	1.64	0.40
	walking down	8.94	0.79
road D	walking up	4.62	1.19
	walking down	8.33	2.11

## Appendix

An indirect Kalman filter for inertial navigation algorithms is introduced in this appendix. The equations are mostly from [10].

Let *q̂*, *r̂*, and *v̂* be the estimates of *q*, *r* and *v*, which are computed from the following equations:

(11) $$\dot{\hat{q}}=\frac{1}{2}\Omega (y_{g})\hat{q}$$

where Ω(*y_g_*) is defined by

(12) $$\begin{array}{l} \Omega (y_{g})=\left[\begin{array}{cccc} 0 & -y_{g,x} & -y_{g,y} & -y_{g,z}\\ y_{g,x} & 0 & y_{g,z} & -y_{g,y}\\ y_{g,y} & -y_{g,z} & 0 & y_{g,x}\\ y_{g,z} & y_{g,y} & -y_{g,x} & 0 \end{array}\right]\\ \;\;\;\;\;\;\;\;\;\;\;\;\;\;\;\;\;\;\;\dot{\hat{v}}=C^{\prime }(\hat{q})y_{a}-\tilde{g}\\ \;\;\;\;\;\;\;\;\;\;\;\;\;\;\;\;\;\;\;\;\dot{\hat{r}}=\hat{v}. \end{array}.$$

where *g͂* is the gravitational acceleration vector.

If there are no sensor noises and bias terms in *y_a_* and *y_g_*, *q̂*, *r̂*, and *v̂* should be 100% accurate. For example, if *y_g_* = *w_b_*, then *q̂* = *q* (that is, there is no error in *q̂*). Due to sensor noises, however, *q̂*, *r̂*, and *v̂* contain errors.

Let *q_e_*, *r_e_*, *v_e_*be errors in *q̂*, *r̂*, and *v̂*, which are defined by

(13) $$\begin{array}{lll} q_{e} & ≜ & \hat{q}*⊗q\\ r_{e} & ≜ & r-\hat{r}\\ v_{e} & ≜ & v-\hat{v} \end{array}$$

where ⊗ is the quaternion multiplication.

If the error *q_e_* is small, it can be approximated by

(14) $$q_{e}\approx \left[\begin{array}{c} 1\\ {\bar{q}}_{e} \end{array}\right].$$

The errors are estimated using a Kalman filter and the state for the Kalman filter is given by

$$x≜\left[\begin{array}{l} {\bar{q}}_{e}\\ b_{g}\\ r_{e}\\ v_{e}\\ b_{a} \end{array}\right].$$

The state equation is given by

(15) $$\dot{x}(t)=Ax(t)+B\left[\begin{array}{c} v_{g}\\ w_{b_{g}}\\ v_{a}\\ w_{b_{a}} \end{array}\right]$$

where *A* and *B* are given by (*I*_3_ ∈ *R*^3×3^ is an identity matrix and 0_3_ ∈ *R*^3×3^ is a zero matrix)

$$A≜\left[\begin{array}{ccccc} \left[-y_{g}\times \right] & -\frac{1}{2}I_{3} & 0_{3} & 0_{3} & 0_{3}\\ 0_{3} & 0_{3} & 0_{3} & 0_{3} & 0_{3}\\ 0_{3} & 0_{3} & 0_{3} & I_{3} & 0_{3}\\ -2C^{\prime }(\hat{q})[y_{a}\times ] & 0_{3} & 0_{3} & 0_{3} & -C^{\prime }(\hat{q})\\ 0_{3} & 0_{3} & 0_{3} & 0_{3} & 0_{3} \end{array}\right]$$

$$B≜\left[\begin{array}{cccc} -0.5 & I_{3} & 0_{3} & I_{3}\\ 0_{3} & I_{3} & 0_{3} & 0_{3}\\ 0_{3} & 0_{3} & 0_{3} & 0_{3}\\ 0_{3} & 0_{3} & -C^{\prime }(\hat{q}) & 0_{3}\\ 0_{3} & 0_{3} & 0_{3} & I_{3} \end{array}\right].$$

For a vector *p* ∈ *R*^3^, [*p*×] is defined by

$$[p\times ]≜\left[\begin{array}{ccc} 0 & -p_{3} & p_{2}\\ p_{3} & 0 & -p_{1}\\ -p_{2} & p_{1} & 0 \end{array}\right].$$

The noise *w_b_g__* and *w_b_a__* are process noises for compensation of slowly time-varying gyroscope and accelerometer bias. As usual, we assume all noises in Equation (15) are uncorrelated, zero mean white Gaussian.

When a foot is on the ground and thus is not moving, we can use the fact *v* = 0 in the measurement update of the Kalman filter. The zero velocity interval can be detected using *y_a_* and *y_g_* : if *y_a_* change is small and *y_g_* is small for more than a certain period, we can consider a foot is not moving [15,19]. The zero velocity interval also can be detected using the force sensors installed on a shoe. In this paper, the zero velocity interval is detected using *y_a_* change and the force sensors.

During the zero velocity interval, we can use the fact *v* = 0 in the measurement updating as follows: The velocity measurement *y_v_* is given by

$$\begin{array}{l} y_{v}=v+v_{v}\\ \;\;\;\;\;\;\;\;\;\;\;\;=\hat{v}+v_{e}+v_{v} \end{array}$$

where *v_v_* is the measurement noise. We assume that the measurement noise *v_v_* is a uncorrelated white Gaussian noise. Let *R_v_* be the covariance of *v_v_**,* which is defined by E{*v_v_*(*t*)*v_v_*(*s*)′} = *R_v_δ*(*t* − *s*).

During the zero velocity interval (that is *v* = 0), we use 0 − *v̂* as an output to the indirect Kalman filter, where the output equation is given by

(16) $$\begin{array}{l} -\hat{v}=v_{e}+v_{v}\\ \;\;\;\;\;\;\;\;\;\;=\left[\begin{array}{ccccc} 0 & 0 & 0 & I_{3} & 0 \end{array}\right]x+v_{v}. \end{array}$$

In the zero velocity updating, *v_v_* is not the actual measurement noise since we are not directly measuring *v* but indirectly estimating *v* = 0. Thus covariance *R_v_* indicates our confidence in the zero velocity algorithm. If we use *R_v_* = 0_3_, we are assuming that the zero velocity algorithm is 100% correct. We note that there is a slight chance that the zero velocity interval detection is wrong. Thus we assign small positive value to *R_v_*. In the zero velocity updating, the velocity, position and attitude errors are greatly reduced [7,8].

During the zero velocity interval, we also update the heading using *y_m_*. As in [7], heading is only compensated at the end of the zero velocity interval since the attitude is most accurate at that time. For the heading update equation, we used the technique in [20], where *y_m_* only affects heading not pitch and roll. See Equation (21) in [20].

The sampling rate of the inertial sensors is 100 Hz and the appropriate discretized equations are used.
